# Restricted cell elongation in *Arabidopsis *hypocotyls is associated with a reduced average pectin esterification level

**DOI:** 10.1186/1471-2229-7-31

**Published:** 2007-06-17

**Authors:** Paul Derbyshire, Maureen C McCann, Keith Roberts

**Affiliations:** 1 Department of Metabolic Biology, John Innes Centre, Colney Lane, Norwich, NR4 7UH, UK; 2 Department of Biological Sciences, Purdue University, West Lafayette, IN 47907, USA; 3 Department of Cell and Developmental Biology, John Innes Centre, Colney Lane, Norwich, NR4 7UH, UK

## Abstract

**Background:**

Cell elongation is mainly limited by the extensibility of the cell wall. Dicotyledonous primary (growing) cell walls contain cellulose, xyloglucan, pectin and proteins, but little is known about how each polymer class contributes to the cell wall mechanical properties that control extensibility.

**Results:**

We present evidence that the degree of pectin methyl-esterification (DE%) limits cell growth, and that a minimum level of about 60% DE is required for normal cell elongation in *Arabidopsis *hypocotyls. When the average DE% falls below this level, as in two gibberellic acid (GA) mutants *ga1-3 *and *gai*, and plants expressing pectin methyl-esterase (*PME1*) from *Aspergillus aculeatus*, then hypocotyl elongation is reduced.

**Conclusion:**

Low average levels of pectin DE% are associated with reduced cell elongation, implicating PMEs, the enzymes that regulate DE%, in the cell elongation process and in responses to GA. At high average DE% other components of the cell wall limit GA-induced growth.

## Background

Young, dividing and expanding cells are surrounded by an extensible primary wall that can allow turgor-driven increases in cell volume. In dicotyledonous plants, primary cell walls are composed of two major interpenetrating polysaccharide networks of cellulose-xyloglucan and pectin, in roughly equal proportions, but the contribution that each polymer class makes to wall extensibility is not yet understood.

The cellulose-xyloglucan network is considered to be the major load-bearing structure [1,2]. Cellulose microfibrils are generally oriented perpendicular to the direction of cell expansion and, because of their tensile strength, define an axis of growth by limiting radial expansion [3]. Breaking and reforming of the xyloglucan chains, that inter-connect cellulose microfibrils, by wall glucanases [4] and xyloglucan-endotransglycosylases (XETs) [5,6], and/or disruption of attachment sites between cellulose and xyloglucan by expansins [7], may then promote longitudinal growth through slippage of the microfibrils. However, little is known about how the surrounding pectin matrix might play a role in this process, either independently or in concert with the cellulose-xyloglucan network. A unique property of pectin is its ability to form gels with varying mechanical strength. Removal of methyl-esters from the pectic galacturonic acid residues by pectin methyl-esterase (PME) [8] creates negatively charged regions of the homogalacturonan (HG) backbone. Depending upon the extent and pattern of de-esterification, these can coordinate with divalent metal ions such as calcium and promote cross-links [9,10], or generate swelling forces through mutual electrostatic repulsion [11]. These two forces exert opposing effects but can have a major influence over the gelling properties of pectin, and a profound effect on wall extensibility. Indeed, the spatial variation in methyl-esterification levels at intercellular spaces suggests that HG has an *in vivo *mechanical role within the cell wall [12] and contributes to the mechanical properties to the wall. Rhamnogalacturonan II-borate di-di-ester cross-links have also been shown to be load-bearing in tensile strength assays of *Arabidopsis *hypocotyls [13].

Methyl, acetyl, phenolic and other unidentified ester linkages in varying proportions represent the ester content of HG, and a relationship between primary wall pectin esterification and cell expansion has been described in a variety of systems. An early study, using ruthenium red to stain negatively charged carboxyl groups of HG, showed the stain was strongest in the basal part of sunflower (*Helianthus annuus*) hypocotyls, where cell elongation had slowed or stopped, whereas further up the hypocotyl, cells continued to elongate and ruthenium red staining was relatively weaker [14]. Similarly, along the axis of mung bean (*Vigna radiata*) hypocotyls, elongating regions have elevated levels of highly methyl-esterified pectins, in contrast to basal regions that have stopped growing and contain fewer esterified HG residues [15]. Highly methyl-esterified regions also have walls that are more plastic, with reduced PME activity, as opposed to mature, stiffer walls at the base of the hypocotyl where PME activity is higher [16]. More recently, direct biochemical analysis in maize (*Zea mays*) showed that total cell wall ester content rises during coleoptile elongation and then falls as growth ceases, but the proportion of methyl-esters is not changed [17]. Similarly, a sharp rise in methyl-esterification occurs when tobacco (*Nicotiana tabaccum*) cell suspension cells elongate, but is at a lower constant level prior to this [18]. The degree of esterification DE% falls in cells that have completed the elongation phase, however, methyl-esters are unchanged and a fall in other esters must account for the reduced DE%. Thus, in tobacco suspensions, methyl-esterification levels may regulate the onset of cell elongation, but are not necessarily involved in cessation of elongation. Likewise, differences in the composition and architecture of type I and type II cell walls [1] may reflect the differing roles that alternative ester groups might play in regulating wall extensibility.

Genetic manipulation of PMEs using over-expression studies has recently allowed the link between DE% and cell expansion to be tested further, but has given more complex results. Potato (*Solanum tuberosum*) plants over-expressing a putative PME from *Petunia inflata *showed increased PME activity in leaves and tubers but did not affect DE%, whereas cell wall ion binding capacity was affected in tubers and yield was reduced [19]. Similarly, antisense inhibition of a putative PME in pea (*Pisum sativum*) roots increased extracellular pH and inhibited root cap border-cell separation leading to stunted root growth, but effects on DE% were not reported [20]. In contrast, expression of an *Aspergillus niger *PME in tobacco reduced the proportion of methyl-esters in pectin and reduced cell size, creating dwarf plants [21]. PMEs therefore appear to have diverse roles in wall metabolism and plant development.

The *Arabidopsis *hypocotyl has been widely used to study the effects of light and hormones on plant growth responses [22,23]. It is also an appropriate system in which to study cell elongation, since it grows almost exclusively by cell expansion and is essentially division-free [24-26]. In this paper, we use two well-characterised gibberellic acid (GA) mutants to identify cell wall compositional changes that may be related to the inhibition of hypocotyl elongation. The GA-deficient *ga1-3 *is a loss of function mutant in the *GA1 *gene which encodes an enzyme involved in GA biosynthesis [27-29]. As a result, *ga1-3 *has reduced amounts of GA [30] and is severely dwarfed, but can be rescued by an exogenous supply of GA [29]. The semi-dominant *gai *mutant has a similar dwarf phenotype to *ga1-3 *but cannot be rescued by exogenous GA [31]. GAI is a member of the DELLA family of putative transcription factors, key components of GA-signalling [32]. GAI and other members of this family (RGA/RGL) act as repressors of plant growth, but are themselves repressed in the presence of endogenous GAs [33,34]. Thus, in *ga1-3 *all DELLA proteins are active. In *gai*, a 17 amino acid deletion in the DELLA region of GAI alters the structure and function of the protein such that it can no longer be repressed by GA [33,35].

Using these two mutants, and particularly the conditional rescue of cell elongation by GA in the *ga1-3 *mutant, we show that active cell elongation is associated with a higher average level of pectin esterification. If DE% is reduced by the over-expression of a well-characterised fungal PME, then cell elongation is decreased.

## Results

### Hypocotyl growth kinetics in two dwarf GA mutants

**Figure 1:**
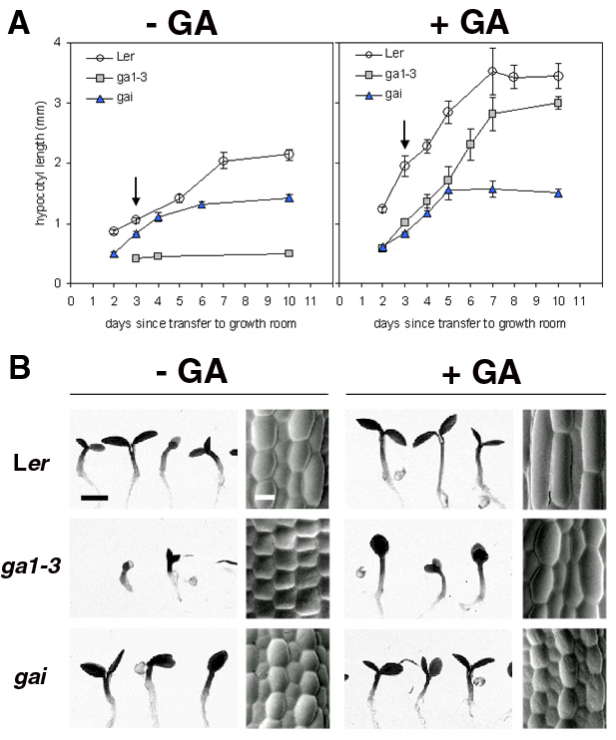
**Growth kinetics and hypocotyl cell elongation in WT (L*er*), *ga1-3*, and *gai *seedlings grown with and without exogenous gibberellic acid (GA)**. (A) Seedlings were grown in continuous light for 10 d with plates in a horizontal position and hypocotyl growth measured over this period. Measurements are an average taken from 5 to 15 seedlings ± SE for each time point. Arrows indicate time (3 d) at which hypocotyls were at approximately 50% of their final length. (B) Light micrographs showing phenotypes of 3-d-old seedlings described in (A) (left panel for each treatment), bar = 1 mm, and FESEM micrographs of hypocotyl epidermis (right panel for each treatment), bar = 25 μm.

*ga1-3 *provides a system in which cell elongation in the hypocotyl can be rescued conditionally by exogenous application of GA, while *gai *provides a control for the effects of exogenous GA application. Hypocotyl growth kinetics in wild-type (WT) (L*er*), *ga1-3*, and *gai *seedlings were established in a continuous light environment with plates positioned horizontally. Hypocotyl growth was measured during a period of 10 d after the culture plates were transferred to the growth room, in the presence and absence of 1 μM exogenous GA_4 _(Figure 1A), a concentration that restores hypocotyl length of *ga1-3 *to WT length [36]. In the absence of exogenous GA, WT hypocotyls elongate between 2 and 7 d, and have a final length of around 2 mm. *ga1-3 *required an extra day to germinate, after which hypocotyl elongation was minimal, reaching only 0.6 mm. *gai *hypocotyls elongate for up to 6 d, but at a slower rate than the WT, with a maximum length of about 1.6 mm. In the presence of exogenous GA, WT hypocotyls elongate between 2 and 7 d, and have final lengths of approximately 3.5 mm, and such hypocotyls grow longer and at a faster rate than without GA. *ga1-3 *hypocotyls respond to exogenous GA, elongating for up to 7 d, with final lengths of around 3 mm. Finally, *gai *does not respond to exogenous GA, having the same hypocotyl growth kinetics and final length as in the absence of the growth regulator, thus confirming its insensitivity to GA. These results are consistent with those reported previously [36]. However, in our analysis, final hypocotyl lengths are shorter, probably as a consequence of the inhibitory effects of the continuous light regime used.

Our analysis of WT, *ga1-3*, and *gai *hypocotyls and their cell walls used material taken at an equivalent developmental stage; in our case defined as approximately 50% of final hypocotyl length, estimated from the growth curves in Figure 1A and indicated by arrows. This was set at 3 d, both in the presence and absence of GA. However, for *ga1-3*, in the absence of GA, hypocotyls barely grow following germination. Therefore we analysed hypocotyls at 3 d, the earliest time point following germination. The general morphology of 3-d-old seedlings of average hypocotyl length is shown in Figure 1B. In the absence of exogenous GA, WT hypocotyls are approximately 1 mm long, but are almost twice as long (1.8 mm) when grown in the presence of exogenous GA. In contrast, *ga1-3 *seedlings are severely dwarfed with hypocotyls at approximately 0.5 mm in length. When grown in the presence of exogenous GA, *ga1-3 *hypocotyl length is restored to that of untreated WT. In the absence of GA, *gai *seedlings have slightly shorter hypocotyls than WT, at about 0.8 mm, and are unaffected by exogenous GA. GA-regulation of hypocotyl growth is mediated through elongation of the pre-existing cells with little or no contribution from cell division [36]. To test whether continuous light affects this process, epidermal cells were imaged with a field-emission scanning electron microscope (FESEM) (Figure 1B). In the absence of exogenous GA, WT epidermal cells are almost twice as long as those of *ga1-3*, while *gai *epidermal cells are slightly shorter than WT. In the presence of exogenous GA, WT epidermal cells approximately double in length, *ga1-3 *epidermal cell length is increased 2 to 3 fold and *gai *epidermal cell length is unchanged. The relative differences in epidermal cell length closely match the relative differences in hypocotyl length. As the same relative differences in cell length have also been observed in the cortical and endodermal layers [37], the differences in hypocotyl length are likely to reflect differences in cell length and therefore in cell elongation.

### Fourier Transform Infrared (FTIR) microspectroscopy of WT and mutant hypocotyls

FTIR microspectroscopy has been used to measure the composition of plant cell walls [38-40]. Small areas of tissue can be selected for analysis, and other advantages include the speed of both sample preparation and data collection. We used FTIR microspectroscopy to quickly ascertain if DE% was associated with dwarfism in primary cell walls of *Arabidopsis *hypocotyls. Spectra were collected from a 200 × 100 μm area in the central region along the length of WT and *ga1-3 *hypocotyls, grown in the presence and absence of exogenous GA and at the developmental stages indicated in Figure 1B. The central stele was avoided to prevent contamination from secondary cell wall components. For each population of hypocotyls, DE% was determined semi-quantitatively based on the method described by Filippov and Kohn [41]. Table 1 shows cell walls of WT hypocotyls have a DE of about 60% when grown both in the presence and absence of GA. In contrast, DE is lowest in walls of *ga1-3 *hypocotyls grown without GA, at about 40%, but rises to around 55% when grown in the presence of GA. Thus, GA-promoted cell elongation in *ga1-3 *hypocotyls is associated with a corresponding rise in DE%.

**Table 1:** Semi-quantitative determination of DE% in WT and *ga1-3 *hypocotyl cell walls.

	semi-quantitative DE%	
	
genotype	no GA	1 μM GA
L*er *(WT)	62.2 ± 1.3	57.1 ± 2.0
*ga1-3*	39.7 ± 2.9	53.4 ± 1.9

DE% was derived from FTIR spectra (*n *= 10 to 28) for each genotype/treatment based on the method of Filippov and Kohn [41]. Average values are given ± SE.

### Biochemical analysis of hypocotyl cell walls

**Figure 2:**
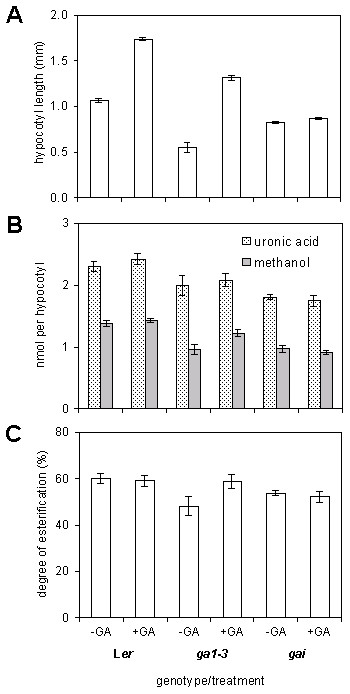
**Effects of gibberellic acid (GA) on degree of esterification (DE%) in WT (L*er*), *ga1-3 *and *gai *hypocotyl cell walls**. (A) Hypocotyl length at time of excision in 3-d-old seedlings. Measurements are an average of 40 to 90 hypocotyls ± SE for each genotype and treatment. (B) Uronic acid content and methyl ester content (measured as methanol) in walls of hypocotyls in (A). Each assay was performed on 50 to 100 hypocotyls for each genotype/treatment and repeated at least once in each experiment. Each experiment was performed three times. Amount of uronic acid and methanol was converted to nmol per hypocotyl in each replicate assay and the total values pooled. Measurements are the average of 6 to 9 replicates ± SE for each genotype and treatment. (C) Degree of methyl-esterification (DE%) in walls of hypocotyls in (A). Values in (B) (including SE) were ratioed (methanol to uronic acid) to give DE%.

To more accurately determine pectin DE%, we measured HG content as uronic acid, and methyl-ester content as the amount of methanol released, at the developmental stages described in Figure 1B. Average hypocotyl lengths used in all experiments are shown in Figure 2A. When grown without exogenous GA, WT (L*er*) hypocotyls measured 1.06 ± 0.02 mm, and increased to 1.74 ± 0.02 mm in the presence of GA. Dwarf *ga1-3 *hypocotyls were 0.55 ± 0.02 mm but increased to 1.31 ± 0.03 mm with exogenous GA. Finally, *gai *hypocotyls measured 0.82 ± 0.01 and 0.86 ± 0.01 mm, when grown without or with GA respectively. Uronic acid and methanol content are expressed as amount per hypocotyl. Since hypocotyl growth is essentially division-free, a change in the amount of a particular wall component can be correlated primarily to cell elongation.

When grown in the absence or presence of GA, WT uronic acid content was 2.31 ± 0.09 and 2.43 ± 0.10 nmol per hypocotyl, respectively, and so was not significantly different between the two treatments (Figure 2B). In *ga1-3 *hypocotyls, grown without GA, the values were lower than WT, measuring 2.00 ± 0.16 nmol per hypocotyl, and was unchanged at 2.10 ± 0.11 nmol per hypocotyl when grown in the presence of GA. *gai *hypocotyls contained the lowest amount of uronic acid, at 1.81 ± 0.04 and 1.75 ± 0.09 nmol per hypocotyl when grown without or with GA respectively. As with WT and *ga1-3*, GA did not affect the uronic content of *gai *hypocotyls. GA also did not significantly affect methanol released in WT. In the absence of GA, methanol released was 1.38 ± 0.04 nmol per hypocotyl. When grown in the presence of exogenous GA, methanol released from WT was 1.44 ± 0.03 nmol per hypocotyl. Therefore, GA affected the amount of neither uronic acid nor methanol released in WT cell walls, even though hypocotyl length increased almost two-fold over the same period of growth. In contrast, GA increased the amount of methanol released from *ga1-3 *cell walls, rising from 0.97 ± 0.08 nmol per hypocotyl in the absence of exogenous GA, to 1.23 ± 0.06 nmol per hypocotyl when grown in the presence of GA. GA-stimulated growth therefore correlates with an increase in cell wall methyl-esterification. Finally, *gai *hypocotyls contained similarly reduced amounts of methanol to *ga1-3*, at 0.97 ± 0.05 nmol per hypocotyl when grown without GA, and was not significantly altered with GA, at 0.91 ± 0.04 nmol per hypocotyl.

The ratio of methanol to uronic acid content was used to calculate DE% (Figure 2C). In WT hypocotyls this was 60.04 ± 2.23% and 59.08 ± 2.31% in the absence and presence of exogenous GA, respectively. GA therefore promotes cell elongation and hypocotyl growth in WT but does not affect DE%. In contrast, GA did affect DE% in *ga1-3 *hypocotyls. In the absence of exogenous GA, DE was 48.23 ± 4.00%, rising to 58.89 ± 3.12% when grown in the presence of GA. GA-stimulated growth in the dwarf *ga1-3 *hypocotyls therefore correlated with the recovery of DE% to WT levels in this mutant. In the semi-dwarf hypocotyls of *gai*, DE was 53.91 ± 1.08 and 52.25 ± 2.52% when grown either without or with GA, respectively. A correlation therefore exists, between hypocotyl length and DE%. The shortest hypocotyls of *ga1-3 *have the lowest DE%, but stimulation of hypocotyl extension by GA also increases DE% to the WT level. *gai *hypocotyl length is intermediary between *ga1-3 *and WT regardless of GA, as is the measured DE% in this mutant.

In summary, an increase in hypocotyl length, and therefore cell elongation, is also accompanied by an increase in DE%. However, enhanced growth of WT induced by GA does not affect DE%. These data suggest that the degree of pectin esterification may affect cell elongation in a GA-deficient and GA-insensitive background.

### Heterologous PME expression reduces hypocotyl length and DE%

To directly test our hypothesis that a low average DE% may constrain growth, we artificially manipulated DE% using reverse genetics. Our prediction would be that reducing the DE% should inhibit hypocotyl elongation. T-DNA insertions into putative PMEs might in principle reduce the potential for de-esterification and ionic cross-linking, leading to an increase in wall extensibility. In *Arabidopsis*, 67 putative PMEs, in Carbohydrate Esterase Family 8, have been identified based on protein sequences [42]. Therefore, the scope for functional redundancy in this family is high, and gene knock-outs might not reveal clear phenotypes. In addition, no PMEs have been biochemically characterised in this species, and some may actually be pectin trans-esterases [43,44]. For the same reasons, homologous over-expression of endogenous or other plant putative PMEs, without biochemical characterisation, may give results that are difficult to interpret [19,20]. In contrast, several *bona fide *PMEs have been reported in bacteria and fungi [45,46]. In *Aspergillus aculeatus*, the *PME1 *gene has been rigorously tested and biochemically characterised [47]. We therefore transformed the *PME1 *cDNA clone into *Arabidopsis *under the control of a constitutive promoter. Interestingly, constitutive expression of *PME1 *yielded no transformants and therefore is probably lethal.

Analysis of the predicted signal peptide region using pSORT showed a low probability of the PME1 protein localising to the cell wall in plants. Therefore, we removed the signal peptide sequence and replaced it with one from a putative PME from *Arabidopsis *(At4g12390) that had a high probability of targeting the protein to the cell wall. The ethanol-inducible expression system was used [48], in which the chimeric construct was cloned downstream of the AlcA promoter, and then transformed into line P5-3 carrying the AlcR promoter. Several independent lines carrying the transgene were identified by PCR using gene-specific primers. To induce expression of the transgene, seedlings were grown for 3 d in continuous light with plates in a near vertical position, and then transferred to induction medium containing 0.1% ethanol in the solidified medium. Transfer at this time point, allowed germination to take place and hypocotyls to enter the rapid phase of elongation. Two lines, PME01 and PME08, in which hypocotyl growth was affected only in the presence of ethanol, were selected for further analysis.

**Figure 3:**
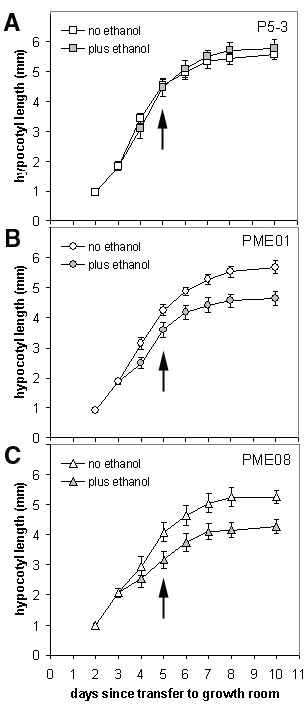
**Growth kinetics and hypocotyl cell elongation in P5-3, PME01, and PME08 seedlings**. Seedlings were grown in continuous light for 10 d with plates in a near vertical position and hypocotyl growth measured over this period. Measurements are an average taken from 12 to 20 seedlings ± SE for each time point. After 3 d seedlings were transferred to control medium, or induction medium containing 0.1% (v/v) ethanol. Arrows indicate time (5 d) at which hypocotyls were further analysed. (A) P5-3, (B) PME01, (C) PME08.

Hypocotyl growth kinetics are shown in Figure 3. In the absence of ethanol, P5-3 hypocotyls grew over a period of 6 d, from day 2 to day 8, with a final length of 5.56 ± 0.17 mm (Figure 3A). The concentration of ethanol used to induce PME1 expression did not affect either the growth profile or final length of P5-3 hypocotyls, which measured 5.77 ± 0.29 mm at day 10. However, compared to previous experiments (Figure 1A), the duration and extent of hypocotyl elongation was increased when plates were positioned vertically, and may be the result of additional nutrient uptake and/or touch responses from being in contact with the surface of the growing medium. In the absence of ethanol, both PME01 and PME08 hypocotyls followed a similar growth profile as P5-3. Final lengths were 5.67 ± 0.22 and 5.25 ± 0.21 mm in lines PME01 and PME08, respectively (Figure 3B, C). However, transfer of the seedlings to induction medium resulted in a deflection of the growth curve for both expressing lines. Hypocotyls stopped growing about 1 d earlier, and final lengths were 4.63 ± 0.24 and 4.27 ± 0.23 mm, respectively, representing a length reduction of about 20%.

**Figure 4:**
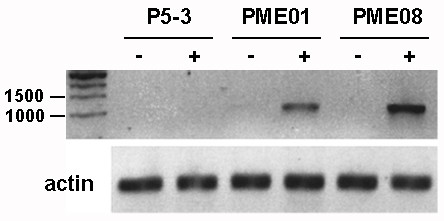
**Transcriptional analysis of *PME1 *using RT-PCR**. RNA was extracted from hypocotyls after 2 d growth on control/induction medium (arrows in Figure 3) and reverse transcribed. *PME1 *expression was detected using gene-specific primers to amplify a 932 bp product. Actin isoform 2-specific primers were used as controls. Lanes denote treatment, (-) no ethanol, and (+) 0.1% ethanol.

Transcriptional and cell wall analysis was performed on excised hypocotyls after 2 d growing on control/induction medium (arrows in Figure 3). At this time point (day 5), the *A. aculeatus *PME was strongly expressed in both lines when grown in the presence of ethanol, whereas no expression was detected in seedlings grown on ethanol-free medium or in P5-3 (Figure 4). Expression was stronger in line PME08 compared to PME01. Both parental lines had reduced seed yield, which may be a consequence of auto-induced *PME1 *expression during seed set, and/or during pollen tetrad separation, the latter involving PME [49]. Thus, it was difficult to collect enough transgenic hypocotyls for direct chemical analysis. Therefore, to confirm that the growth effects were due to pectin de-esterification, we again used FTIR microspectroscopy of individual hypocotyls to measure DE% indirectly (Table 2). At this time point, hypocotyl lengths in P5-3 were 4.52 ± 0.19 and 4.46 ± 0.30 mm when grown in the absence and presence of ethanol, respectively. In the absence of ethanol, PME01 hypocotyls were 4.25 ± 0.19 mm long, compared to 3.60 ± 0.24 mm when grown on induction medium. Similarly, PME08 hypocotyls were 4.08 ± 0.33 and 3.15 ± 0.29 mm after 2 d growth on control and induction medium, respectively. Induced expression of *PME1 *therefore corresponded to a 15% reduction in average hypocotyl length in line PME01, and a 22% reduction in line PME08, compared to non-induced seedlings. DE in P5-3 hypocotyls was about 48% in the absence of ethanol, and about 45% in the presence (Table 2). In line PME01, DE was about 48% in the absence of ethanol, but only about 40% following induction. In line PME08, DE was about 42% in the absence of ethanol, and reduced to about 38% when induced. The overall reduction in DE in P5-3, from about 60% (Table 1) to about 48% (Table 2), may be due to the slowing down of hypocotyl elongation at day 5, as opposed to day 3 when they are growing fastest. Nevertheless, the lowest DE% we measured, in both lines, followed *PME1 *induction. In summary, *PME1 *expression corresponded to a reduction in cell wall DE% and hypocotyl length in both lines. Expression was strongest in line PME08 in which we measured both the lowest DE% and the shortest hypocotyls.

**Table 2:** Semi-quantitative determination of DE% in P5-3, PME01 and PME08 hypocotyl cell walls.

	semi-quantitative DE%	
	
genotype	no ethanol	0.1% ethanol
P5-3	48.6 ± 1.0	44.9 ± 1.2
PME01	47.7 ± 1.5	40.2 ± 2.0
PME08	42.2 ± 1.2	38.5 ± 1.2

Hypocotyls were prepared after 2 d growing on control (no ethanol) or induction (0.1% ethanol) medium. DE% was derived from FTIR spectra (*n *= 20 to 21) for each genotype/treatment as described in Table 1. Average values are given ± SE.

## Discussion

In this work, we used the hypocotyl of the *ga1-3 *mutant, as a system in which we can induce cell elongation, to investigate the relationship between the level of pectin esterification and cell elongation. We measured low DE% in this dwarf GA-deficient mutant, and a high average DE% in WT hypocotyl cell walls. Intermediate DE% between *ga1-3 *and WT were found in the GA-insensitive mutant *gai *that correlated with its semi-dwarf hypocotyl, and GA-induced growth of *ga1-3 *was paralleled by a recovery of DE% to WT. However, further increases in WT hypocotyl growth, induced by GA, were not accompanied by further changes in DE% above the maximum. This suggests that a permissive level of DE% exists in the primary cell wall of *Arabidopsis *hypocotyls, and that a reduction in average DE% below this level progressively reduces cell elongation. Above this level, other factors become limiting for growth. Reducing DE%, by alcohol-induced expression of *PME1 *from *A. aculeatus*, resulted in a predicted inhibition of hypocotyl growth. Since endogenous PMEs are responsible for the removal of methyl-esters from cell wall pectin, we predict that one or more members of this family of enzymes plays a role in regulating cell elongation *in vivo*.

Pectin is synthesised and deposited in the wall in a highly methyl-esterified form [50], with measurements as high as 80% DE [17,18]. In *Arabidopsis *hypocotyls we measured maximal DE of ~60% (Figure 2C), and, it is likely that pectin is synthesised at values above this and subsequently de-esterified to a level where it is maintained. At this level, pectin may be at the optimal DE% to contribute to wall plasticity and thus to cell elongation, but de-esterification to levels below this progressively restricts plasticity and hence hypocotyl growth. Current theories of how DE% may regulate wall extensibility, and thus cell expansion, are largely based on *in vitro *studies of pectin gels. Pectin has a highly complex macromolecular structure, and its properties can be modulated by several factors that include pH, osmolarity and ionic conditions [11]. One of the main influences of DE% is regulating the amount of ionised stretches of the HG backbone that can cross-link with calcium ions [9]. A reduction in DE% increases the potential for such cross-links and leads to a more rigid gel with increased visco-elastic properties [12,51]. This may independently affect the extensibility of the cell wall, but may also act by modifying the mechanical properties of the key load-bearing polymers, the cellulose-xyloglucan network. The presence of pectin increases the extensibility, and reduces the stiffness, of cellulose-pectin composites, compared to cellulose alone, with low DE systems (30%) having a greater effect than high DE systems (67%) [52]. Therefore, if wall extensibility is indeed affected by the physico-mechanical properties imposed by DE%, these effects may be autonomous to the pectin network. Indeed, linear stretching experiments show that the pectin network moves independently of the cellulose-xyloglucan network [53,54].

Plant PMEs are thought to remove methyl-ester groups in a blockwise fashion, leading to contiguous stretches of free carboxyl residues within the HG backbone, whereas fungal PMEs are thought to de-esterify pectin randomly resulting in single carboxyl residues that are dispersed throughout the HG portion of pectin [55,56]. The resulting pattern of de-esterification can have different effects on pectin properties. Blockwise de-esterification favours cross-linking [9], requiring at least 9 contiguous carboxyl residues for coordination with calcium [57]. In contrast, random de-esterification may promote swelling, and reduces wall porosity [12]. *In vitro *studies have been performed on calcium-pectin gels with similar DE% but de-esterified either by plant or by fungal PMEs. Gels prepared from fungal PME-treated pectin have no capacity to recover under compression, whereas they recover completely when de-esterified by plant PMEs [12]. Both mode and extent of de-esterification can therefore influence the rheological properties of pectin, and can potentially regulate wall extensibility but by different mechanisms. At an optimum pH of 4.6, *PME1 *is highly effective at de-esterification, removing 75–85% of methyl groups *in vitro *[47]. However, in our study it is unlikely that *PME1 *had a major impact on DE% in hypocotyl cell walls, since indirect measurements showed only modest reductions, i.e., from about 48% to 40% in PME01, and about 42% to 38% in PME08 (Table 2). This may be the result of duration of expression, sub-optimal wall pH and/or accessibility to HG within the cell wall matrix. Therefore, expression of *PME1 *from *A. aculeatus *may have resulted in random de-esterification and affected wall loosening properties more through a reduction in pore space, possibly caused by electrostatic repulsion of fixed negative charges, leading to swelling of the pectin network and more efficient filling of the available spaces [11,58], and reduced porosity may subsequently limit accessibility of wall loosening proteins to their cellulose-xyloglucan substrate. Similarly, inhibition of hypocotyl elongation in *ga1-3 *and *gai *may be due to cross-linking of the pectin network giving stiffer walls, with less effect on pore space. It is important to recognise that we are looking at small effects with this experimental system. High levels of PME are likely to be lethal, and low levels, coupled with random patterns of de-esterification, are likely to have small effects. Nevertheless the tight correlation of extension with DE% is clear. Further studies of the loss- and gain-of-function mutants described here may help to identify any differences in pectin structure that are the result of GA-deficiency/insensitivity, compared to effects of *PME1 *expression.

Since we do not know exactly which polymers are affected by PME1, or where, it is important to consider that small changes in some crucially located pectin molecules may underlie the effects we measured. One possibility is that middle-lamella pectin, which in general is highly de-esterified, may act as a trans-cellular brake, helping coordinate differential growth between adjacent cells to achieve even growth in the organ as a whole [57]. Another possibility, reflecting our awareness that it is probably just the outer epidermal wall that both drives and constrains growth of the hypocotyl [59], is that the pectin in this very thick outer wall [60] alone is involved in the relationship between growth and pectin DE%.

Other studies in which plant PMEs have been constitutively over-expressed have given more complex results. In pea, inhibiting the expression of a PME altered cell wall pH and inhibited the loss of root cap border cells, resulting in swollen roots and reduced elongation [20]. More recently, over-expression of a *Petunia inflata *PME in potatoes caused a transient increase in stem elongation in regions with reduced PME activity [19]. According to the authors, the reduction in PME activity may have been caused by compensation for the effects of over-expression, however down-regulation of PME and increase in stem elongation is consistent with the hypothesis presented here. Neither of these putative PMEs, or indeed any other plant PMEs, have been characterised biochemically so their mechanistic effects on growth remain speculative. In contrast, *PME1 *has been functionally characterised [47], and the inducible system we used [48] gave tight control over its expression. Likewise, a reduction in DE% and production of dwarf tobacco plants resulted when a functionally characterised PME from *Aspergillus niger *was over-expressed [21], further emphasising the need for more rigorous characterisation of these plant enzymes prior to their manipulation. Over-expression of plant-derived PMEs in plants may also be compromised by the presence of endogenous PME inhibitors (PMEIs), a recently identified family of proteins that adds another regulatory level to pectin metabolism and DE% [61-63]. Indeed, over-expression of PMEIs in *Arabidopsis *resulted in a decrease in overall PME activity coupled with an increase in DE%. Transgenic seedlings, consistent with our hypothesis, also produced longer roots and had longer cells in the elongation zone of the root [64].

While GA promoted elongation in WT hypocotyls, it did so with no net increase in cell wall uronic acid content over the same growth period (Figure 2B). Elongation in this case correlates with cell wall thinning [60]. Maintaining DE% at an adequate level may therefore contribute to the strength of the thinning wall, as well as to its extensibility. Similarly, GA-recovery of hypocotyl growth and DE% in *ga1-3 *does not increase net uronic acid content of the dwarf hypocotyl. Taken together, our data suggests that GA also promotes cell elongation via remodelling of the existing wall. Putative wall loosening proteins have been shown to be GA-regulated. For example, GA enhances cell expansion and glucanase activity in maize leaves [65] and wheat (*Triticum aestivum*) internodes [66], and an XET is GA-regulated in germinating tomato (*Lycopersicon esculentum*) seedlings [67]. This correlates with increases in wall extensibility that are not seen in GA-insensitive wheat cultivars [66,68]. GA also increases wall extensibility in lettuce (*Lactuca sativa*) [69] and cucumber (*Cucumis sativus*) hypocotyls [70]. Therefore, in *Arabidopsis *hypocotyls, GA may also promote cell elongation by loosening of the cellulose-xyloglucan network in conjunction with wall remodelling, and restrict it by modulating DE%. In lettuce hypocotyls [71], oat (*Avena sativa*) [72] and wheat internodes [66], both net cell wall polysaccharide and organ elongation are simultaneously increased by GA. Thus, synthesis and deposition versus remodelling of the cell wall during GA-stimulated cell expansion may vary, depending upon the plant species. Relative to WT hypocotyls, uronic acid content was reduced in *ga1-3 *and lowest in *gai*. Therefore, both *GA1 *and *GAI *are required for normal uronic acid incorporation into the wall, as well as for controlling its methyl-ester content.

## Conclusion

We have shown a consistent relationship between the average degree of cell wall pectin esterification (DE%) and the degree of cell elongation in *Arabidopsis *hypocotyls. A reduction in hypocotyl length, using either forward or reverse genetic approaches, is associated with a reduction in DE%. Endogenous PMEs and their inhibitors, which regulate the DE%, are therefore implicated in cell elongation in this system. GA has no effect on DE% in WT hypocotyls but promotes additional cell elongation, suggesting that enzymes regulating the cellulose-xyloglucan network and other components of the primary cell wall may be involved in responses to the growth regulator.

## Methods

### Plant materials and growth conditions

*Arabidopsis thaliana *(L. Heynh) ecotype Landsberg *erecta *(L*er*) was used as the reference wild-type (WT). In the over-expression experiment, line P5-3 (also in the L*er *background) was used as WT. Seeds were surface-sterilised by immersion for 5 min in 5% (v/v) Vortex bleach (Procter & Gamble Ltd, containing 5 to 15% chlorine-based bleach), and washed three times in sterile distilled water (sdH_2_O). Following sterilisation, to allow seeds of *ga1-3 *to germinate, they were incubated at 4°C for 5 d in a solution of 1 μM GA_4 _(Sigma-Aldrich, UK) [36]. L*er *and *gai *do not require this treatment but were included for consistency. Next, seeds were rinsed five times in sdH_2_O and sown onto medium containing 1× Murashige and Skoog (MS) basal salts (micro and macro elements) (Duchefa) supplemented with 3% (w/v) sucrose (pH adjusted to 5.7) and solidified with 0.5% (w/v) Phytagel™ (Sigma-Aldrich, UK). Approximately 20 seeds were evenly sown per 9 cm Petri plate (Bibby Sterilin Ltd) containing 20 mL of growing medium, and plates sealed with Parafilm^® ^laboratory film (Pechiney Plastic Packaging, Menasha, USA). Plates were placed in darkness at 4°C for 48 h to stimulate and synchronise germination. Following cold treatment, plates were transferred to a growth room maintained at 25°C and incubated horizontally under fluorescent lamps (70 μmol m^-2 ^s^-1^) in a continuous white light regime.

### Hypocotyl measurements

Hypocotyl length was determined as the distance between the top of the collet root hairs, to the 'V' made by the cotyledon shoulder [73]. Hypocotyl lengths were measured using a Leica WILD M10 binocular microscope fitted with an eye-piece graticule, and the mean ± SE calculated for each data set.

### Field emission scanning electron microscopy (FESEM)

Seedlings were mounted in a horizontal position on adhesive carbon tabs (Agar Scientific Ltd) and plunge-frozen at -210°C in liquid nitrogen slush. After freezing, samples were immediately loaded into the cryo chamber of the scanning electron microscope, equilibrated with the stage and sublimed at -100°C for 2 min. The temperature was returned to -110°C, the samples were sputter-coated with platinum for 2 min at 10 mA, and then transferred to the imaging stage at -130 to -150°C for analysis. FESEM images of hypocotyl epidermal cells were obtained using a Philips XL30 FEG scanning electron microscope (FEI Co., Eindhoven, The Netherlands) fitted with a cryostage (CT1500 HF; Oxford Instruments, Abingdon, Oxford, UK), operating at 3 kV and a working distance of between 5 and 15 mm.

### FTIR microspectroscopy

Whole hypocotyls were excised from seedlings and suspended on the surface of water-soaked tissue paper to prevent tissue dehydration during sample collection. This also effectively rinsed the samples. The samples were compressed onto barium fluoride (BaF_2_) windows (13 × 2 mm) (Crystran Ltd, Poole, UK), dried at 60°C for 1 h and used immediately for spectral acquisition, or stored overnight at 4°C and used the next day. Windows were supported on the stage of a UMA500 microscope accessory of a Bio-Rad FTS175c spectrometer equipped with a liquid nitrogen-cooled mercury cadmium telluride detector and absorbance spectra obtained. Sixty-four interferograms were collected in transmission mode with 8 cm^-1 ^resolution and co-added to improve the signal-to-noise-ratio for each sample. An area of approximately 200 × 100 μm in the middle region (along the longitudinal axis) of each hypocotyl was selected, avoiding the central stele. One spectrum was collected from each hypocotyl and between 10 and 28 samples for each genotype/treatment used. For each population the spectra were averaged between 790 and 1810 cm^-1 ^and each average spectrum baseline-corrected and area-normalised to account for differences in sample thickness. Processing of spectral data was done using OMNIC E.S.P. 5.0 software. For each spectrum, a two-point baseline was constructed between 870 and 1810 cm^-1^. The absorbance maxima of bands *υ*_as_(COO^-^) 1605 cm^-1 ^and *υ*(C = O)_ester _1745 cm^-1 ^from the baseline were measured, and the log ratio of these values used to semi-quantitatively calculate DE% from the calibration curve of Filippov and Kohn [41]. For each genotype/treatment, values were averaged ± SE.

### Uronic acid and methyl ester assays

Hypocotyls were excised precisely using fine-tipped forceps and a razor blade. Upon excision, samples were transferred to a 1.7 mL microfuge tube containing 1 mL absolute ethanol and heated to 85°C for 20 min to extract chlorophyll, sugars and other small molecules. An additional extraction was made in 1 mL 80% (v/v) ethanol at 85°C for a further 20 min, and then rinsed three times in 1 mL sdH_2_O. Samples were suspended in a small volume of sdH_2_O and freeze-dried. Each tube contained between 50 and 100 hypocotyls. Uronic acid assays were performed on these as described previously [74]. Methyl-esters were determined as the amount of methanol released following saponification using the method described by Kim and Carpita [17]. Values are expressed as nmol per hypocotyl. For each genotype and treatment, duplicate or triplicate samples were used in each experiment, and each experiment performed three times. In total, 600–900 hypocotyls were used to independently calculate average uronic acid and average methanol values. The ratio of methanol to uronic acid was used to calculate DE%. Thus in total, between 1200 and 1800 hypocotyls were used to derive the average DE% for each genotype/treatment. Standard error values were ratioed as described previously [75].

### Construction of plasmids and plant transformation

The open reading frame of *PME1 *(Accession no: U49378) from *Aspergillus aculeatus *[47], minus the predicted signal peptide sequence, was PCR amplified out of pYES 2.0 using the forward primer OVEXP3 (5'-CTGCCAATCCACCATAGCCGCCAGCCGTACCACGGCTCC-3') and the reverse primer OVEXP4 (5'-GGCGAATTCTTTAATTAGAAGTAGGAGGTATCGAC-3'). The underlined region denotes the *Eco*RI restriction site. The signal peptide sequence of a putative PME (At4g12390) from *Arabidopsis *was PCR amplified from BAC clone T4C9 (supplied by ABRC) using the forward primer OVEXP1 (5'-GGCGGATCCTTATGGAACCAAAGCTAACCCA-3') and the reverse primer OVEXP2 (5'-GGAGCCGTGGTACGGCTGGCGGCTATGGTGGATTGGCAG-3'). The underlined region denotes the *Bam*HI restriction site. The plant signal peptide sequence was ligated to the fungal PME sequence giving a 1133 bp cDNA product, and then digested with *Bam*HI/*Eco*RI and ligated into pL4 upstream of the AlcA35S promoter and downstream of CaMV35S terminator. The vector was linearised by digesting with *Bgl*II, followed by a second digestion with *Hin*dIII to give a 1696 bp fragment containing the AlcA35S::PME::CaMV35S terminator region. The gel-purified product was ligated into pGreen0229 using *Hin*dIII/*Bam*HI and the chimeric construct transformed via *Agrobacterium tumefaciens *(GV3101) into line P5-3 (containing the ethanol-inducible AlcR promoter) using the floral-dip method [76]. Tranformants were selected with Basta and T2 plants used for phenotypic analysis.

### Plant growth and ethanol induction

Seeds were prepared as described above and sown onto sterile filter paper in contact with growing medium containing 1% (w/v) sucrose. Sealed plates were incubated in a near vertical position. This allowed hypocotyls to be measured each day without opening plates, which would have resulted in some loss of ethanol vapour (see below). After 3 d seedlings were carefully transferred to the same medium containing no ethanol (control medium) or to induction medium containing 0.1% (v/v) ethanol. Induction medium was prepared by adding the appropriate volume of 50% (v/v) of ethanol to the molten medium cooled just to the point at which it started to solidify in order to prevent ethanol evaporation. Following transfer, plates were resealed with Parafilm. Hypocotyl lengths were imaged digitally and measured using Photoshop 5.0 software.

### Transcription analysis by RT-PCR

RNA was extracted from whole seedlings at 2 d after transfer to induction/control medium, using a QIAGEN RNeasy Plant minikit according to the manufacturer's instructions. RNA yield was quantified by spectrophotometry and concentrations equalised with RNase-free water. After DNase treatment (40 units DNaseI; Amersham Pharmacia) for 20 min at 37°C, 2.5 μg was reverse transcribed for 60 min at 42°C in a final volume of 20 μL in the presence of 20 units RNA guard, 1 mM dNTPs, 5 mM MgCl_2_, 0.3 μM oligo(dT) primers and 4 units M-MLV reverse transcriptase (Life Technologies) in the reaction buffer provided. Reactions were stopped by heat inactivation and 80 μL H_2_O added. 2 μL of the reverse transcription reaction were used for PCR amplification. The forward primer PMEfor (5'-GTACCACGGCTCCCTCCG-3') and the reverse primer PMErev (5'-GTAGGAGGTATCGACCCAGC-3') gave a 932 bp product for the transgene cDNA. The forward primer Actin2-5' (5'-CTAAGCTCTCAAGATCAAAGGCTTA-3') and the reverse primer Actin2-3' (5'-ACTAAAACGCAAAACGAAAGCGGTT-3') amplified a 220 bp fragment of *ACT2 *cDNA and used as a semi-quantitative control [77]. For controls, 25 cycles of PCR were conducted (30 s at 94°C, 30 s at 55°C, 1 min at 72°C) in a final volume of 20 μL containing 2 μL cDNA, 1 mM dNTPs, 5 mM MgCl_2_, 0.3 μM Actin forward/Actin reverse primers and 0.5 units of Taq DNA polymerase (Life Technologies) in the reaction buffer provided. For quantification of the *PME1 *transgene 30 cycles of PCR were conducted as described above using PMEfor/PMErev primers. The latter reaction was also used to confirm presence of the transgene following Basta selection.

## Authors' contributions

PD conducted all of the experiments and wrote drafts of the manuscript. MCM helped supervise the project. PD, MCM and KR co-wrote the manuscript. KR oversaw the project in his lab and is the guarantor of the work.
